# Gut Microbiota and Fecal Microbiota Transplantation in Patients with Food Allergies: A Systematic Review

**DOI:** 10.3390/microorganisms10101904

**Published:** 2022-09-26

**Authors:** Caroline Jensen, Marie Fagervik Antonsen, Gülen Arslan Lied

**Affiliations:** 1 Centre for Nutrition, Department of Clinical Medicine, University of Bergen, 5021 Bergen, Norway; 2 Division of Gastroenterology, Department of Medicine, Haukeland University Hospital, 5020 Bergen, Norway; 3 Section of Clinical Allergy, Department of Occupational Medicine, Haukeland University Hospital, 5020 Bergen, Norway

**Keywords:** food allergy, gut microbiota, fecal microbiota transplantation

## Abstract

The prevalence of food allergies (FAs) has increased considerably in recent decades, with the only available treatment being the avoidance of the specific food items causing the allergy. FAs may have a major impact on quality of life, and it is of great interest to explore new strategies to prevent and treat FAs. Some studies show an altered gut microbiota profile in individuals with FAs, and the modulation of gut microbiota is therefore proposed as a potential strategy for prevention and treatment. This systematic review aimed to investigate: (1) the gut microbiota profile in individuals with FAs compared to healthy individuals and (2) the effect of fecal microbiota transplantation (FMT) on gut microbiota profiles and/or allergy symptoms. A literature search was conducted in PubMed (Medline) on 5 April 2022. Of the 236 publications identified, 12 studies were included based on inclusion and exclusion criteria. Eleven of these studies reported results on the gut microbiota in children with FAs compared to healthy controls (HCs). The majority of studies (six studies) observed no difference in alpha diversity when comparing children with FAs to HCs; however, a difference in beta diversity was observed in five studies. At the phylum level, we observed a high abundance of Firmicutes (six studies) and Proteobacteria (five studies), whereas a low abundance of Bacteroidetes (5 studies) was observed in children with FAs compared to HCs. Of the 12 included studies, four explored the effect of FMT on gut microbiota and/or allergy symptoms. Three studies reported that transferring gut microbiota from children without FAs to germ-free mice, protected the mice against allergic reactions, whereas one study did not report findings on the allergic symptoms. The results on gut microbiota after FMT varied and were too divergent to draw any conclusions. Overall, our results suggest that there are differences in the gut microbiota profile in individuals with FAs compared to individuals without FAs. FMT seems to be a promising strategy to prevent allergic symptoms but needs to be further explored in animal and human models. As the findings in this review are based on a small number of studies (12 studies), further studies are warranted before any clear conclusions can be drawn regarding gut microbiota profiles and the effect of FMT on individuals with FAs.

## 1. Introduction

Humans have a community of micro-organisms, including bacteria, viruses and eukaryotes, living inside their digestive tract known as gut microbiota [1,2]. These microbes are of great importance to health as they are involved in several physiological processes in the human body, such as protection from infectious agents [2], the biosynthesis of vitamins [3] and fermentation of undigested nutrients [4]. Intestinal gut microbiota is dominated by two bacterial phyla: Firmicutes and Bacteroidetes, followed by Actinobacteria and Verrucomicrobia [5]. There are large interindividual variations in the composition of intestinal gut microbiota, affected by factors such as age, diet, disease and genetics [2]. Despite these variations, a high bacterial diversity and large variation of bacteria in the intestines are linked to good health [6]. A disruption of the stable system in the gut may cause an imbalance in the intestinal microbiota with reduced bacterial diversity, commonly referred to as dysbiosis [2]. Studies have suggested that this may contribute to the development of disorders such as asthma, celiac disease and allergies [7]. 

A food allergy (FA) is an immunological response to a specific allergen in a food resulting in an allergic reaction [8]. Based on the specific immunological mechanism involved, FAs can be classified into three groups: immunoglobulin E (IgE)-mediated allergies, non-IgE-mediated allergies or mixed IgE and non-IgE-mediated FAs. IgE-mediated FAs are the most common in Western countries, with the highest prevalence reported in children under the age of 3 years [8]. Allergies against cows’ milk, eggs, tree nuts, shellfish, wheat, soybeans, peanuts and fish are common IgE-mediated FAs. [9]. Globally, milk and egg allergies are reported as the two most common FAs, whereas the third depends on the geographical location, e.g., peanut allergies in the United States, wheat in Germany and Japan and sesame in Israel [10]. The prevalence of FAs has increased during the last decade, and estimates suggest that as many as 25% of adults in Western countries are reported to have an FA [8]. However, when FAs are diagnosed through testing, such as an oral food challenge, the prevalence estimates reported are much lower, with around 2% of adults and 8% of young children reported to have an FA [8]. A combination of behavioral, socioeconomic and environmental factors, including the typical Western lifestyle, diet and living environment, are believed to be involved in the observed increase in FAs [11,12,13]. 

The primary treatment of FAs involves a strict elimination and avoidance of foods containing the specific allergen [14]. Depending on the type of FA, this may impact diet quality and result in a less diverse diet [14]. An impaired quality of life is commonly reported, particularly in individuals with a combination of several different FAs and in those with a higher risk of potential life-threatening anaphylactic reactions [15]. Therefore, it is of great interest to find new strategies to prevent and treat FAs. The modulation of gut microbiota has been proposed as a potential strategy, and several therapeutic methods are suggested, such as probiotics, prebiotics, synbiotics and fecal microbiota transplantation (FMT) [12]. FMT is a promising medical treatment involving the transfer of gut microbiota from a healthy donor to a recipient [15]. This results in changes in the gut microbiota of the recipient, potentially improving symptoms and eliminating disease [16]. The method is used as treatment for patients with Clostridium difficile colitis [16], showing positive effects in the short- and long-term [17]. Few studies have explored the effect of FMT in allergies; however, there are some promising results from animal studies [12]. Observational studies have found that the risk of developing FAs may be affected by microbial exposure and colonization in early childhood [12]. There is also increasing evidence that the composition of gut microbiota and the diversity of individuals with FAs differ compared to those without FAs [12]. However, there is a need for further investigation, and to the best of our knowledge, few studies have explored the relationship between gut microbiota, FAs and FMT. Therefore, the overall aim of this systematic review was to explore the gut microbiota profile of individuals with FAs compared to healthy controls (HCs) and the effect of FMT on the gut microbiota profile of individuals with FAs. 

## 2. Materials and Methods

The checklist and flowchart of the Preferred Reporting Items for Systematic Reviews and Meta-Analyses (PRISMA) guidelines were followed in this systematic review [18].

### Search Strategy and Criteria for Inclusion

A literature search was conducted in PubMed (Medline) on 5 April 2022, using the search words “Gastrointestinal microbiome”, “Food Hypersensitivity” and “Faecal Microbiota transplantation” as MeSH (medical subject headings) terms. To exclude nonrelevant publications, the search excluded publications with the word “lactose intolerance”, “atopic dermatitis”, “irritable bowel syndrome”, “irritable bowel disease”, “autoimmune disease”, “celiac disease”, “gluten sensitivity”, “diabetes”, “asthma”, “cancer”, “colitis”, “ADHD”, “autism” and “atopic dermatitis”. No search filters were applied. The criteria for inclusion and exclusion are shown in Table 1. 

## 3. Results

### 3.1. Literature Search and Study Characteristics

**Figure 1:**
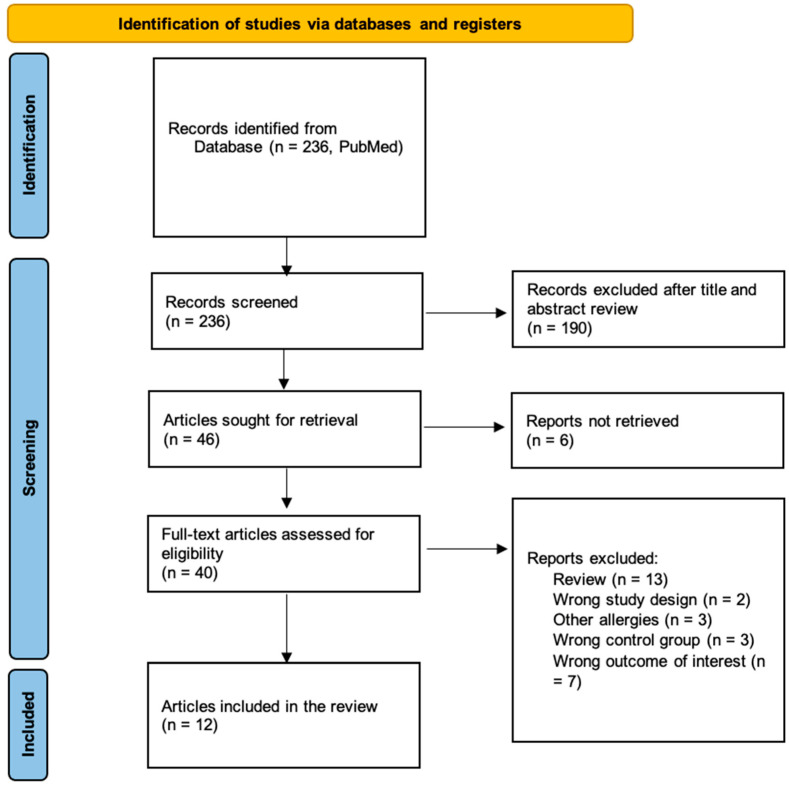
PRISMA (Preferred Reporting Items for Systematic Reviews and Meta-Analyses) flow diagram [18] depicting the literature search in PubMed (Medline) and the screening process for this systematic review.

A literature search was conducted in PubMed (Medline) on 5 April 2022. The search identified 236 articles in total, of which 12 publications were included after full-text reading based on the inclusion and exclusion criteria. Figure 1 illustrates a flow diagram of the selection process. Eleven of the twelve studies reported results on gut microbiota in individuals with FAs compared to an HCs group Four of the twelve studies reported results on gut microbiota and/or allergic symptoms after FMT from human donors (individuals with FAs or HCs) to mice.

### 3.2. Study Characteristics

The study characteristics for the 12 included articles are found in Table 2 and Table 3. Table 2 summarizes the study characteristics of the eleven studies reporting the gut microbiota profile in children with FAs vs. an HC group, whereas Table 3 summarizes study characteristics of the four studies reporting the results on the effect of FMT from human donors (the FA or HC group) to mice on gut microbiota and/or allergic symptoms. The included studies were conducted between 2014–2021. Three of the included studies were conducted in North America [19,20,21], two in Europe [22,23], one in the Middle East [24], five in East Asia [25,26,27,28,29] and one in the United States/Italy [30]. 

All studies collected fecal samples and used 16S rRNA sequencing for analysis of the gut microbiota (intestinal microbiota). To assess the diversity changes, alpha diversity (α-diversity) and/or beta diversity (β-diversity) were used. Alpha diversity describes the diversity of species within a given sample (richness and/or evenness), whereas β-diversity describes the similarities or dissimilarities between two communities [31,32]. To assess α-diversity, the studies used the Shannon index, the Simpson index, Pileou’s evenness and observed species, whereas Unifrac or Bray–Curtis dissimilarity were used to assess β-diversity.

#### 3.2.1. Characteristics of Studies Exploring the Gut Microbiota Profile in Humans with Food Allergies Versus Healthy Controls

Of the 12 included articles, 11 studies reported results on gut microbiota differences between children with FAs compared to an HC group [19,20,21,22,23,24,25,26,27,28,29]. All participants were under the age of 18 years, with most being infants. The total number of participants was 995, with a ratio of 397:444 (female:male). Abdel-Gadir et al. [19] did not report gender differences but divided the study population into different age groups. The participants included in this study were between the age of 1–15 months, and they explored differences in the gut microbiota between the age groups and compared to the age-matched HC groups [19]. 

The participants in the included studies either had an FA or were considered an HC. Kourosh et al. [21] also included nonallergic siblings (n = 25) of the children with FAs. Overall, the ratio of individuals with FAs to HCs was 470:494 (excluding the sibling group in the study by Kourosh et al. [21]). The children with FA either had a cows’ milk, egg, peanut, tree nut, sesame, soy, wheat, fish or shellfish allergy, or a combination of these. The cows’ milk protein allergy (CMA) was the most studied FA among the included studies. The studies greatly differed in whether they included or excluded children based on the type of FA (an IgE- or non-IgE-mediated FA). Most studies required a positive skin prick test or blood test and/or a positive oral food challenge test. All studies excluded children who had used antibiotics and pre- or probiotics during the weeks before fecal sampling. The mode of delivery varied, but some studies reported that children born prematurely were excluded [19,21,26,28]. Four studies excluded children with chronic inflammatory diseases, autoimmune diseases and metabolic diseases [19,22,24,28]. Dong et al. [25] only included breastfed infants, whereas others included formula-fed [28] or both [19,20,21,22,23,26,27]. Two studies did not report any information about feeding methods [24,29]. 

#### 3.2.2. Characteristics of Studies Exploring the Effect of FMT from Human Donors to Mice on Gut Microbiota and/or Allergic Symptoms

Four studies explored the effect of FMT on the gut microbiota and/or allergic symptoms of mice, using infants as fecal donors [19,23,28,30]. In all four studies, they used germ-free mice as the recipients of fecal microbiota from infant donors. The donors were either children with an FA or an HC. Wang et al. [28] matched the donors by age, whereas Feehley et al. [30] and Mauras et al. [23] also matched by gender and mode of delivery. Abdel-Gadir et al. [19] did not report any matching. In all studies, FMT was administered using oral or intragastric gavage. Only Feehley et al. [30] explored changes in the gut microbiota after FMT, with no oral food challenge. Abdel-Gadir et al. [19] only reported the response to an oral food challenge after FMT, with no reports of changes in the gut bacteria. 

### 3.3. Main Findings

The main findings are listed in Table 4 and Table 5. Table 4 shows the results for differences in gut microbiota between children with FAs and HCs (11 articles). Table 5 describes the changes in gut microbiota and/or allergic symptoms after FMT, comparing mice who received microbiota from a donor with FA or an HC (four articles). 

#### 3.3.1. Differences in Gut Microbiota Diversity

When exploring diversity, six of the eleven studies reporting results on gut microbiota in children with FAs did not observe any differences in α-diversity compared to the HC groups [19,20,21,22,26,27]. Four studies reported a decrease in α-diversity in children with FAs compared to the HC groups [24,25,28,29]. One study did not investigate or report any findings on α-diversity [23]. Five studies reported a difference in β-diversity [22,24,25,28,29] in those with FAs, with two studies observing no difference in β-diversity when comparing the two groups [19,20]. Additionally, two studies reported a difference in general diversity [23,25]. Ling et al. [27] reported a lower number of observed operational taxonomic units (OTUs) among the children with FAs, referring to a low richness in different species. 

#### 3.3.2. Differences in Phylum and at the Lower Taxonomic Level

At the phylum level, six studies reported an increase in the level of Firmicutes in children with FAs when compared to HCs [21,22,23,24,26,27]. Two studies reported a decrease [20,29], and one study reported no difference [25] in the level of Firmicutes when comparing children with FAs to HCs. Two of the studies did not report any results on Firmicutes [19,28]. For Bacteroidetes, five studies observed a decrease [20,24,25,27,28], and one study [22] reported an increase when comparing children with FAs to HCs. The other studies did not report any findings on this phylum [19,21,23,26,29]. In the Proteobacteria, five studies showed an increase in children with FAs vs. HCs [20,25,26,28,29]. The other studies did not report any findings. Two studies reported a decrease in Actinobacteria [23,27] in children with FA compared to HCs. The other studies reported no findings on this particular phylum. 

Abdel-Gadir et al. [19] reported differences in species from the *Clostridial* families when comparing children with FAs to HCs, which was also evident for specific age groups. They also reported compositional differences among 77 OTUs within specific age groups of those with FAs compared to HCs. For the OTU50, whose closest reference species is the *Subdoligranulum variabile*, differences occurred in several age groups [19]. Azad et al. [20] observed an increase in the family *Enterobacteriaceae* and decreases in *Bacteroidaceae*, resulting in an increased *Enterobacteriaceae/Bacteroidaceae* ratio (E/B ratio) among the participants with FAs. They also found a decreased level of *Ruminococcaceae*.

Berni Canani et al. [22] reported a higher abundance of the genera *Bacteroides* and *Alistipes*, belonging to the phylum Bacteroidetes, in children with FAs. The genus *Sarcina* from the phylum Firmicutes was also elevated among the children with FAs compared to children without FAs [22]. Dong et al. [25] reported a decrease in *Bacteroides (Bacteroidetes)* and an increase in *Enterobacteriaceae (Proteobacteria)* at the genus level, resulting in an elevated E/B ratio in children with FAs [25].

Goldberg et al. [24] observed a great variability regarding specific bacteria. At the family level, *Enterobacteriaceae* were reduced, and *Erysipelotrichaceae* were elevated in children with FAs compared to HCs. The genera *Adlercreutzia*, Eggerthella and *Turicibacter* were higher, whereas *Enterococcus* was decreased in children with FAs when compared to HCs. They also observed differences at the species level when comparing children with FAs to the HC group (please see Table 4) [24]. Inoue et al. [26] found a higher abundance of *Lachnospira*, *Veillonella* and *Sutterella* and a reduced abundance of *Dorea* and *Akkermansia* at the genus level in children with FAs compared to the HC group.

Kourosh et al. [21] explored the differences between children with FAs and the HC group as well as with their nonallergic siblings. For the children with FAs, the order of Pasteurellales and the species *Haemophilus parainfluenzae* and *Blautia* were decreased compared to HCs. *Oscillibacter valericigenes*, *Lachnoclostridium bolteae* and *Faecalibacterium* were increased in children with FAs compared to HCs and nonallergic siblings. The sibling group differed from those with FAs and the HC group with the high abundance of the species *Alistipes putredinis, Alistipes* and *Odoribacter splanchnicus*. Compared to the HC group, they also observed a low abundance of *Haemophilus parainfluenzae* and *Blautia* in the sibling group [21].

A higher abundance of the phyla Firmicutes and Fusobacteria and a lower abundance of Bacteroidetes, Proteobacteria, Actinobacteria and Verrucomicrobia were observed by Ling et al. [27]. The genera *Lactobacillus, Prevotella* and *Clostridium sensu stricto* were increased, while *Bacteroides, Veillonella* and *Blautia* decreased [27]. More detailed results can be found in Table 4. Mauras et al. [23] showed a low *Bifidobacterium/Lachnospiraceae ratio* in the FA group. A higher abundance of the genus *Eisenbergiella* was also observed [23]. Wang et al. [28] reported elevated *Enterobacteriaceae* and lower *Bacteroidaceae*, resulting in an elevated E/B ratio in the FA group. Yamagishi et al. [29] observed a higher abundance in the order of Enterobacteriales and a lower abundance in Lactobacillales in children with FAs compared to the HC group. This was the only study that explored children with an egg allergy separately [29].

#### 3.3.3. Fecal Microbiota Transplantation and the Effect on Allergy Symptoms and Gut Microbiota

Four out of the twelve articles reported results on the effect of FMT in mice [19,23,28,30]. The results are summarized in Table 5. Three studies explored the response to an oral food challenge or after oral allergic sensitization in the different recipients [19,23,30]. Abdel-Gadir et al. [19] reported a rapid and sustained drop in the core body temperature in mice receiving FMT from infants with FAs, indicating anaphylaxis, whereas only a mild and temporary drop in core body temperature was observed for mice receiving microbiota from healthy donors. Feehley et al. [30] observed that mice receiving gut microbiota from HCs showed no indication of anaphylactic responses. For the FA group, a drop in core body temperature was observed, indicating anaphylaxis and an allergic reaction [30]. Mauras et al. [23] observed symptoms such as diarrhea, scratching and puffiness, common in allergic reactions, as well as a higher fecal score (softer to diarrheic stool/anal inflammation) in mice receiving microbiota from children with FAs (CMA) compared to sensitized control mice receiving microbiota from the healthy control children. No difference in fecal scores were reported in sensitized control mice compared to nonsensitized control mice, with both receiving microbiota from healthy donors, indicating that healthy microbiota may be protective when exposed to an allergen [23]. Wang et al. [28] did not report results from an oral food challenge. They observed a lower α-diversity in mice receiving FMT from an FA donor and found differences in β-diversity between mice receiving FMT from a donor with FAs vs. a healthy donor. For bacterial changes, they observed that the families *Bacteroidaceae* and *Lachnospiraceae* decreased and *Clostridiaceae 1*, *Enterobacteriaceae* and *Bifidobacteriaceae* increased in mice receiving gut microbiota from an FA donor compared to a healthy donor. The genera *Bifidobacterium*, unclassified family *Enterobacteriaceae*, *Raoultella* and *Clostridium sensu stricto* increased, while *Lactobacillus* decreased [28].

Feehley et al. [30] observed no difference in diversity and evenness of gut microbiota but found a lower protective or nonprotective OTU ratio in the mice receiving gut microbiota from a donor with FAs. They also found a lower abundance of the family *Lachnospiraceae* and the species *Anaerostipes caccae* [30]. Mauras et al. [23] reported a lower *Bifidobacteria/Lachnospiraceae* ratio including a decrease in *Anaerostipes* in the mice receiving FMT from an FA donor. Abdel-Gadir et al. [19] differed from the other FMT trials and included interventions with the oral administration of six *Clostridiales* species. They observed that this type of bacteria protected against FAs. This did not involve FMT but only treatment with some specific species [19]. 

## 4. Discussion

Based on a small number of studies, this systematic review has observed that the gut microbiota profile in children with FAs is different compared to individuals without FAs. A high abundance of Firmicutes [21,22,23,24,26,27] and Proteobacteria [20,25,26,28,29] and a low abundance of Bacteroidetes [20,24,25,27,28] seem to be related to the gut microbiota profile of children with FAs. Six studies reported no difference in α-diversity [19,20,21,22,26,27] but a difference in β-diversity [22,24,25,28,29] in children with FAs compared to those without FAs. Based on only four studies, we observed that gut microbiota from a healthy donor protected experimental mice from developing allergic reactions and reflected the microbiota of its donor, indicating that FMT may have potential as a treatment strategy in FAs. The results must be interpreted with caution as they are based on a very small number of studies. No clear conclusion can be drawn, and there is a great need to explore the findings further in both animal models and human studies. 

Overall, more differences were observed in the phyla Firmicutes, followed by Bacteroidetes, Proteobacteria and then Actinobacteria. As not all studies reported differences in all phyla, the results are somewhat difficult to compare. Regardless of the level of taxonomy studied, differences in specific bacteria between children with FAs and those without FAs were observed. At the phylum level, five studies observed a lower abundance of Bacteroidetes [20,24,25,27,28]. Only one study [22] reported an increase in Bacteroidetes in children with FAs. This study differed from the others as it was the only study conducted in Europe. Differences in diet might therefore explain the divergent results, as the participants were past weaning age. A low abundance of Bacteroidetes has been associated with FAs in other studies [33], supporting our results. However, as five of the studies did not report any findings on this phylum [19,21,23,26,29], further studies are needed.

A high abundance of Firmicutes among children with FAs were reported in six studies [21,22,23,24,26,27]. Two studies reported a decrease [20,29], and one study reported no difference [25], whereas two studies did not report any results on Firmicutes [19,28]. The two studies in our review reporting a decrease both examined children with an egg allergy [20,29]. The similar results could therefore be related to the specific allergy, as it has been suggested that the type of allergy may reflect gut microbiota composition [9,11]. Previous studies have also reported a lower abundance of Firmicutes in children with FAs compared to HCs [34,35,36], which conflicts with most of the studies in our review which report a higher abundance. The different findings may be due to factors such as the type of allergy, age, differences in ethnicity and diet. It is suggested that Firmicutes may have a protective effect on the development of FAs, with a high abundance of Firmicutes and increased bacterial diversity being associated with the resolution of CMA by the age of 8 years [35]. Three studies reported an increased E/B ratio among children with FAs compared to those without FAs [19,24,27], indicating that a high E/B ratio (high Proteobacteria, low Firmicutes) might be associated with FAs. This supports findings suggesting the protective effect of Firmicutes [19,35]. 

An increase in Proteobacteria among children with FAs compared to those without FAs was reported in five of the included studies [20,25,26,28,29], similar to previous results [37]. Four of these studies were conducted in East Asia [25,26,28,29], suggesting that ethnicity and diet may impact the findings. However, one study was conducted in Canada [20], therefore not supporting this theory. The age of participants differed from three months to four years, indicating that the maturation of the gut microbiota may not be related to the similarities observed in gut microbiota. This is surprising, considering that gut microbiota evolves tremendously during the infant years [38], with a major development of gut microbiota beginning at the weaning age (approximately 6 months old) [38]. However, it may be other factors, such as diet, ethnicity or the type of FA, that have a bigger impact and are the reason for the similar results between studies. 

The studies exploring FMT all reported similar results. Three of the four studies reported that transferring gut microbiota from children without FAs to germ-free mice protected the mice against allergic reactions [19,23,30]. Abdel-Gadir et al. [19] observed a mild drop in the core body temperature of mice receiving fecal matter from healthy children, with a higher drop in body temperature in mice receiving gut microbiota from children with FAs, which was indicative of an anaphylactic response in the latter group. When it comes to gut microbiota composition and diversity, there were divergent results. One study reported no difference in microbiota diversity and richness in mice receiving FMT from children with or without FAs [30], supporting some of the included studies which did not perform FMT [19,20]. On the other hand, a lower α-diversity and differences in β-diversity in mice receiving FMT from children with FAs compared to HCs were reported by Wang et al. [28], which is consistent with other studies [24,25,28,29]. Overall, there seems to be some promising effect of using FMT from healthy donors in protecting mice from developing allergic reactions. However, the results are based on a very limited number of studies and in animal models. The studies also reported varied results on microbiota composition and diversity, making it difficult to draw any conclusion. Overall, there is a need to further explore these findings, both in animal models and human studies. 

There are several reasons that could explain the inconsistent findings in the current review. Firstly, the role of gut microbiota and use of FMT in FAs is a relatively new field, and there is a limited number of existing articles with varying quality. The results must be carefully interpreted as a low number of studies was included in this review. Secondly, the included studies differed in study design and methodology. The majority used case–control, cohort or a cross-sectional design, with some studies not clearly reporting the study design. These differences may have impacted our results. Further, the recruitment of participants might also be of importance. Some studies excluded participants with a history of atopic manifestations other than FAs, for example, children with both FAs and atopic dermatitis, which applies to many children with FAs. In addition, the number of participants, age, ethnicity and different diets of the participants may have influenced the results. The number of participants varied from 6 to 291, which arguably influences the power of the results. The mode of delivery and if the children were breast- or formula-fed is another factor that may have affected the results. The differences in gut microbiota have been observed comparing breast-fed and formula-fed infants [38]. However, the feeding strategy was similar between the studies, and they did match for breastfeeding and the mode of delivery. It is therefore assumed that this did not have any major impact on the results. The type of FA studied also varied between studies, making it difficult to compare studies. Our results could also have been strengthened by separating the results into specific FAs as it is suggested that different FAs may impact gut microbiota composition and diversity differently [9,11]. This was not achieved due to the small number of included studies. Another limitation is the comparison between the different phyla. When looking at the phylum level, the bacteria at lower taxonomic levels may be left out. Some studies reported both increases and decreases at e.g., the species level, but when looking at the overall results, they observed that the prevalence in the phylum pointed in one direction. This indicates the importance of exploring lower taxonomic levels to increase our understanding. However, there was a large variation between the studies at lower levels. In general, it is well-known that there are large individual variations in gut microbiota [38], making it difficult to compare results across studies. 

## 5. Conclusions

In conclusion, the results from this review suggest that there is a difference in the gut microbiota profile between individuals with FAs compared to individuals without FAs. A high abundance of Firmicutes and Proteobacteria and low abundance of Bacteroidetes seem to be related to the gut microbiota profile of children with FAs. The results on Firmicutes are somewhat conflicting with previous studies, and there is a need to further explore these findings. Further, FMT seems to be a promising strategy for the prevention and/or treatment of FAs as the included studies observed that FMT from a healthy donor protected mice from developing allergic symptoms. As the findings in this systematic review are based on a small number of studies, further studies are warranted before any clear conclusions may be drawn regarding the gut microbiota profile of individuals with FAs and the effect of FMT.

## Figures and Tables

**Table 1:** Inclusion and exclusion criteria.

Criteria	Inclusion	Exclusion
Study design	Observational studies	Review
	Intervention studies (both RCTs, nonrandomized and noncontrolled studies)	Meta-analysis Systematic review
Population	Individuals with food allergies	No food allergy ^1^
	HC group	No HC group
	Children (<18 years)	Adults (>18 years)
Outcome	Changes in gut microbiota (diversity/composition)	Reporting changes in gut microbiota after dietary intervention ^2^
	Changes in gut microbiota/allergic symptoms after FMT from human donors (FA/HC) to mice or humans	No FMT/FMT from other donors
Language	English	Other languages
	Full text available	No full text available

^1^ Studies not defining whether they explored a food allergy or a respiratory allergy were excluded. ^2^ Studies exploring the effect of dietary intervention, probiotics or other interventions on gut microbiota were excluded. Abbreviations: FA, food allergy; FMT, fecal microbiota transplantation; and HC, healthy control; RCT: randomized controlled trial.

**Table 2:** Characteristics of the studies exploring gut microbiota in individuals with food allergies vs. healthy controls.

First Author, Year of Publication	Study Design	Country	Sample Size (Female:Male)	Age (Months ± SD)	Groups	Allergies	Gut Microbiota Protocol
Abdel-Gadir, 2019 [19]	Observational study ^1^	USA	154 (N/A)	1–15 (all)	FA (n = 56) HC (n = 98)	Milk, soy, eggs, tree nuts, fish, shellfish, wheat, peanuts	Fecal samples
							16S rRNA
							α- and β-diversity ^2,3^
Azad, 2015 [20]	Longitudinal cohort study	Canada	166 (81:85)	11.8 ± 0.8 (all)	FA (n = 12) HC (n = 154)	Eggs, peanuts	Fecal samples
							16S rRNA
							α- and β-diversity ^2,3^
Berni Canani, 2018 [22]	Cross-sectional study	Italy	52 (31:21)	11.4 ± 7.2 (FA) 12.9 ± 7.4 (HC)	FA (n = 23) HC (n = 23)	Non-IgE CMA	Fecal samples
							16S rRNA
							α- and β-diversity ^2,3^
Dong, 2018 [25]	Case–control follow-up study	China	120 (56:64)	2.9 ± 1.0 (all)	FA (n = 60) HC (n = 60)	CMA	Fecal samples
							16S rRNA
							α- and β-diversity ^2,3^
Goldberg, 2020 [24]	Observational study ^1^	Israel	291 (120:171)	77 (63.0–114.5) ^4^ (FA) 78 (48.0–125.3) ^4^ (HC)	FA (n = 233) HC (n = 58)	Milk, peanuts, sesame, tree nuts	Fecal samples
							16S rRNA
							α- and β-diversity ^2,3^
Inoue, 2017 [26]	Preliminary study	Japan	8 (4:4)	48 ± 21.166 (all)	FA (n = 4) HC (n = 4)	Eggs, wheat, soybeans, sesame, milk, peanuts, shellfish	Fecal samples
							16S rRNA
							α- and β-diversity ^2,3^
Kourosh, 2018 [21]	Case–control study	USA	68 (36:32)	>7 years (n = 26) 7–18 years (n = 42)	FA (n = 22) HC (n = 21) NFA-S (n = 25)	Peanuts, tree nuts, fish, milk, eggs, sesame, soy	Fecal samples
							16S rRNA
							α-diversity ^2^
Ling, 2014 [27]	Observational study ^1^	China	79 (40:39)	5.8 ± 1.83 (IgE) 5.0 ± 1.37 (non-IgE) 5.6 ± 2.33 (HC)	FA (n = 34) HC (n = 45)	Milk, soybeans, eggs, wheat, shrimp, tree nuts, fish, peanuts	Fecal samples
							16S rRNA
							α- and β-diversity ^2,3^
Mauras, 2019 [23]	Observational study ^1^	United Kingdom	11 (8:3)	9.77 ± 3.38 (all)	FA (n = 5) HC (n = 6)	CMA	Fecal samples
							16S rRNA
							α-diversity ^2^
Wang, 2021 [28]	Observational study ^1^	China	6 (6:0)	3.33 ± 1.15 (HC) 3.67 ± 1.15 (FA)	FA (n = 3) HC (n = 3)	CMA	Fecal samples
							16S rRNA
							α- and β-diversity ^2,3^
Yamagishi, 2021 [29]	Observational study ^1^	Japan	40 (15:25)	3.1 years (FA) 4.0 years (HC)	FA (n = 18) HC (n = 22)	Eggs	Fecal samples
							16S rRNA
							α- and β-diversity ^2,3^

^1^ Study design not reported; ^2^ α-diversity: Shannon diversity index, Simpson index, observed species, evenness index, Faith’s phylogenetic diversity (PD), abundance-based coverage estimator (ACE) and Chao1 index; ^3^ β-diversity: UniFrac, Jaccard distance and Bray–Curtis dissimilarity. ^4^ Interquartile range. Abbreviations: CMA, cows’ milk protein allergy; FA, food allergy; HC, healthy control; and NFA-S, nonfood allergic sibling.

**Table 3:** Characteristics of studies exploring fecal microbiota transplantation (FMT) in mice from human donors.

First Author, Year of Publication	Country	Sample Size, Mice:Donor	Recipients (Mice)	Donor	Sensitization Subject	Oral Challenge	FMT Protocol	Gut Microbiota Protocol
Abdel-Gadir, 2019 [19]	USA	14:N/A	Adult GF	FA and HC infants	OVA/SEB on the skin and via oral gavage (daily for 1 week)	OVA	FMT from FA or HC infants ^1^.	Fecal samples
				Matched for age				N/A
								N/A
Feehley, 2019 [30]	Italy/USA	73:8	GF From FA (n = 42) From HC (n = 31)	FA (n = 4) HC (n = 4)	BLG and CT	BLG	Intragastric gavage with infant fecal homogenate (CMA or HC).	Fecal samples
								16S rRNA
				Matched for age, gender and mode of delivery				α- and β-diversity ^2,3^
Mauras, 2019 [23]	United Kingdom	N/A:2	Three-week-old GF	FA (n = 1) HC (n = 1)	NS (n = 12–13): CT S (n = 5–6): WP and CT	BLG after last sensitization	Oral gavage (from FA or HC)	Fecal samples (1st, 3rd and 5th sensitization) 16S rRNA α-diversity ^2^
				Matched for age, gender and mode of delivery	Once a week for 5 weeks			
Wang, 2021 [28]	China	11:6	Six-week-old GF mice From FA (n = 6) From HC (n = 5)	FA (n = 3) HC (n = 3)	N/A	N/A	Oral gavage	Fecal samples (14 days post-FMT)
				Matched for age				16S rRNA
								α- and β-diversity ^2,3^

^1^ Method not reported. ^2^ α-diversity: Shannon index, Simpson index, Pielou’s evenness and observed species. ^3^ Unifrac and Bray–Curtis. Abbreviations: N/A, not available; OVA, ovalbumin; SEB, staphylococcal enterotoxin B; FA, food allergy; HC, healthy control; GF, germ-free; BLG, β-lactoglobulin; CT, cholera toxin; WP, whey protein; NS, nonsensitized; S, sensitized; and FMT, fecal microbiota transplantation.

**Table 4:** Results from studies exploring gut microbiota in children with food allergies compared to healthy controls.

First Author, Year of Publication	Allergy	FA vs. HC (Diversity and E/B Ratio)	Taxonomic Rank	GM Composition (FA vs. HC)
Abdel-Gadir, 2019 [19]	Milk, soy, eggs, tree nuts, fish, shellfish, wheat, peanuts	N/D α- and β-diversity	Species	Differences in Clostridiales ^1^ Difference in 77 OTUs Difference in *Subdoligranulum variabile* (OTU50 ^2^)
Azad, 2015 [20]	Milk, eggs, peanuts	N/D α- and β-diversity ↑ E/B ratio	Phylum	↓ Bacteroidetes
			Family	↑ *Enterobacteriaceae* ↓ *Bacteroidaceae* ↓ *Ruminococcaceae*
Berni Canani, 2018 [22]	Non-IgE CMA	N/D α-diversity	Phylum	↑ Bacteroidetes
			Genus	↑ *Bacteroides* ↑ *Alistipes* ↑*Sarcina*
Dong, 2018 [25]	CMA	↓ α-diversity ↑ E/B ratio Difference in β-diversity	Phylum	↓ Bacteroidetes ↑ Proteobaceria
			Family	↑ *Enterobacteriaceae* ↓ *Bacteroidaceae*
			Genus	↓ *Bacteroides*
Goldberg, 2020 [24]	Milk, tree nuts, peanuts, sesame	↓ α-diversity Difference in β-diversity	Family	↑ *Erysipelotrichaceae* ↓ *Enterobacteriaceae*
			Genus	↑ *Adlercreutzia* ↑ *Eggerthella* ↑ *Turicibacter* ↓ *Enterococcus*
			Species	↑ *Collinsella aerofaciens* ↑ *Dorea formicigenerans* ↑*Blautia obeum* ↓*Prevotella copri* ↓*Bifidobacterium adolescentis*
Inoue, 2017 [26]	Milk, soy, eggs, wheat, shellfish, peanuts, sesame	N/D α-diversity	Genus	↓ *Dorea* ↓ *Akkermansia* ↑ *Lachnospira* ↑ *Veillonella* ↑ *Sutterella*
Kourosh, 2018 [21]	Milk, soy, eggs, tree nuts, fish, peanuts, sesame	N/D α-diversity N/D OTUs observed	Order	↓ *Pasteurellales*
			Species	↑ *Oscillibacter valericigenes* ↑ *Lachnoclostridium bolteae* ↑ *Faecalibacterium* ↓ *Haemophilus parainfluenzae* ↓ *Blautia*
			Species	Sibling group ↑ *Alistipes putredinis* ↑ *Alistipes sp.* ↑ *Odoribacter splanchnicus* ↓ *Haemophilus parainfluenzae* ↓ *Blautia (Genus)*
Ling, 2014 [27]	Milk, soy, eggs, tree nuts, fish, shellfish, wheat, peanuts	N/D α-diversity	Phylum	↑ Firmicutes ↑ Fusobacteria ↓ Bacteroidetes ↓ Proteobacteria ↓ Actinobacteria ↓ Verrucomicrobia
			Family	↑ *Clostridiacea 1* ↑ *Cytophagaceae* ↑ *Nocardiaceae*
			Genus	↑ *Clostridium sensu stricto (IgE)* ↑*Enterococcus* ↑*Lactobacillus* ↑*Bifidobacterium* ↑*Staphylococcus* ↑*Faecalibacterium* ↑*Clostridium XIVa* ↑*Anaerostipes* ↑*Prevotella* ↑*Clostridium XVIII (non-IgE)* ↑*Flavonifractor* ↓*Bacteroides (non-IgE)* ↓*Veillonella* ↓*Blautia* ↓*Clostridium XI* ↓*Lachnospiraceae incertae sedis (non-IgE)*
Mauras, 2019 [23]	CMA	↑ Bacterial diversity	Family	↑ *Lachnospiraceae*
			Genus	↓ *Bifidobacterium* ↑ *Eisenbergiella*
Wang, 2021 [28]	CM-FPIAP (Non-IgE)	↑ E/B ratio ↓ α-diversity	Family	↑ *Enterobacteriaceae* ↓ *Bacteroidaceae*
Yamagishi, 2021 [29]	Eggs	↓ α-diversity Difference in β-diversity ↓ OTUs observed	Order	↑ *Enterobacteriales* ↓ *Lactobacillales*

An arrow pointing down (↓) are referring to a decrease/reduction, whereas an arrow pointing upwards (↑) are referring to an increase. ^1^ In specific age groups. Clusters I, IV, XI and XIVa. ^2^ Variable as closest reference. Abbreviations: N/A, not available; N/D, no difference, CM-FPIAP, cows’ milk food protein-induced allergic proctocolitis; CMA, cows’ milk protein allergy; and OTU, operational taxonomic unit.

**Table 5:** Results from studies exploring changes in gut microbiota and/or allergic symptoms after fecal microbiota transplantation in mice receiving microbiota from a donor with food allergies or a healthy control.

First Author, Year of Publication	Differences FA vs. HC	Responses to OAS and/or OFC (HC Mice)	Responses to OAS and/or OFC (FA Mice)	Bacterial Changes
Abdel-Gadir, 2019 [19]	OA of six *Clostridiales* species protected against FA (not FMT)	↓ (mild) Core body temperature	↓ Core body temperature	N/A
Feehley, 2019 [30]	N/D Community diversity and evenness ↓ protective/nonprotective OTU ratio	Protected from anaphylactic responses	↓ Core body temperature	Family: ↓ *Lachnospiraceae* Species: ↓ *Anaerostipes caccae*
Mauras, 2019 [23]	↓ *Bifidobacteria*/*Lachnospiraceae* ratio	Protected from allergic reactions	Diarrhea-related symptoms ↑ Clinical scores ^1^	Family: ↑ *Lachnospiraceae* Genus: ↓ *Bifidobacteria* ↓ *Anaerostipes*
Wang, 2021 [28]	↓ α-diversity Difference β-diversity	N/A	N/A	Family: ↓ *Bacteroidaceae* ↓ *Lachnospiraceae* ↑ *Clostridiaceae* 1 ↑ *Enterobacteriaceae* ↑ *Bifidobacteriaceae* Genus: ↑ *Bifidobacterium* ↑ unclassified family *Enterobacteriaceae* ↑ *Raoultella* ↑ *Clostridium sensu stricto* ↓ *Lactobacillus*

An arrow pointing down (↓) are referring to a decrease/reduction, whereas an arrow pointing upwards (↑) are referring to an increase. ^1^ Scratching, puffiness, loss of mobility. Abbreviations: N/D, no difference; N/A, not available; OA, oral administration; OAS, oral allergic sensitization; OFC, oral food challenge; OTU, operational taxonomic unit; HC, healthy control; and FA, food allergy.
